# P-19. Identification of Risk Factors for Mortality in Pseudomonas aeruginosa Bacteremia: What Can We Modify?

**DOI:** 10.1093/ofid/ofaf695.250

**Published:** 2026-01-11

**Authors:** Maximiliano Gabriel Castro, Nicolás Rearte, Candelaria Del Carril, Fabián Herrera, Natalin Grippo, Elena Temporiti, Diego Torres, Marcia Querci, Agustina Fiori, Nicolás Lasserre, Gustavo A Castro Torres, Julia Ramponi, Pablo Bonvehí

**Affiliations:** CEMIC: Centro de Educacion Medica e Investigaciones Clinicas Norberto Quirno, Ciudad autónoma de Buenos Aires, Ciudad Autonoma de Buenos Aires, Argentina; CEMIC: Centro de Educacion Medica e Investigaciones Clinicas Norberto Quirno, Ciudad autónoma de Buenos Aires, Ciudad Autonoma de Buenos Aires, Argentina; CEMIC: Centro de Educacion Medica e Investigaciones Clinicas Norberto Quirno, Ciudad autónoma de Buenos Aires, Ciudad Autonoma de Buenos Aires, Argentina; Centro de Educación Médica e Investigaciones Clínicas, CEMIC, BUENOS AIRES, Ciudad Autonoma de Buenos Aires, Argentina; Hospital Universitario CEMIC, Buenos Aires, Ciudad Autonoma de Buenos Aires, Argentina; Hospital Universitario CEMIC, Buenos Aires, Ciudad Autonoma de Buenos Aires, Argentina; Centro de Educación Médica e Investigaciones Clínicas, CEMIC, BUENOS AIRES, Ciudad Autonoma de Buenos Aires, Argentina; Hospital Universitario CEMIC, Buenos Aires, Ciudad Autonoma de Buenos Aires, Argentina; CEMIC: Centro de Educacion Medica e Investigaciones Clinicas Norberto Quirno, Ciudad autónoma de Buenos Aires, Ciudad Autonoma de Buenos Aires, Argentina; CEMIC: Centro de Educacion Medica e Investigaciones Clinicas Norberto Quirno, Ciudad autónoma de Buenos Aires, Ciudad Autonoma de Buenos Aires, Argentina; CEMIC: Centro de Educacion Medica e Investigaciones Clinicas Norberto Quirno, Ciudad autónoma de Buenos Aires, Ciudad Autonoma de Buenos Aires, Argentina; CEMIC: Centro de Educacion Medica e Investigaciones Clinicas Norberto Quirno, Ciudad autónoma de Buenos Aires, Ciudad Autonoma de Buenos Aires, Argentina; CEMIC: Centro de Educacion Medica e Investigaciones Clinicas Norberto Quirno, Ciudad autónoma de Buenos Aires, Ciudad Autonoma de Buenos Aires, Argentina

## Abstract

**Background:**

*Pseudomonas aeruginosa* bacteremia (PAB) is associated with a high mortality rate, and many factors can contribute to poor outcomes. We aimed to identify risk factors for attributable mortality of PAB episodes.

**Methods:**

A retrospective cohort study of hospitalized patients diagnosed with PAB between June 2014 and December 2024 was performed. Epidemiological, clinical characteristics and outcomes were described. A multivariate logistic regression analysis identified independent risk factors associated with 30-day attributable mortality (RFM).

**Results:**

124 episodes were included, with a median age of 66.5 years (IQR 52.3–74) and a median Charlson comorbidity index score of 4 (IQR 2–7). The median time from hospital admission to bacteremia onset was 12 days (IQR 0–22), and 69.4% of patients had received antibiotics before the bacteremia episode. Only 3 cases of difficult-to-treat resistant PA isolates were detected; however, 28.2% were resistant to carbapenems. The respiratory tract was the most common source of infection (32.3%), followed by the urinary tract (20.9%). A total of 51.6% of patients required intensive care unit admission, 41.9% developed shock, and 51.6% multiorgan failure. Empirical antibiotic therapy was appropriate in 83.1% of cases; nevertheless, 30-day overall mortality was 37.9%, and attributable mortality of PAB accounted for 80.9% of these deaths. RFM were hematologic malignancy [OR 4.28 (95% CI 1.09-16.8, p=0.03)], respiratory source [OR 10.8 (95% CI 3.00-39, p< 0.001)], shock [OR 30.5 (95% CI 6.76-137, p< 0.001)] and carbapenem resistance [OR 17.3 (95% CI 3.65-82.2, p< 0.001)].

**Conclusion:**

Carbapenem-resistant strain was the second most significant risk factor for attributable mortality. This finding highlights the potential impact of robust antimicrobial stewardship and infection control programs for reducing the mortality associated with these infections.

**Disclosures:**

All Authors: No reported disclosures

